# Selective retina therapy and thermal stimulation of the retina: different regenerative properties - implications for AMD therapy

**DOI:** 10.1186/s12886-021-02188-8

**Published:** 2021-11-30

**Authors:** Elisabeth Richert, Julia Papenkort, Claus von der Burchard, Alexa Klettner, Philipp Arnold, Ralph Lucius, Ralf Brinkmann, Carsten Framme, Johann Roider, Jan Tode

**Affiliations:** 1 Department of Ophthalmology, Christian-Albrechts-University of Kiel, University Medical Center, Kiel, Germany; 2 Friedrich-Alexander-University Erlangen-Nürnberg, Nürnberg, Germany; 3 Christian-Albrechts-University of Kiel, Institute of Anatomy, Kiel, Germany; 4 Medical Laser Center Lübeck, Lübeck, Germany; 5 Institute for Biomedical Optics, University of Lübeck, Lübeck, Germany; 6 Department of Ophthalmology, Hannover Medical School, Carl-Neuberg-Str. 1, 30625 Hannover, Germany

**Keywords:** Selective retina therapy (SRT), Thermal stimulation of the retina (TSR), Age- related macular degeneration (AMD), Regeneration, Rejuvenation

## Abstract

**Background:**

Selective Retina Therapy (SRT), a photodisruptive micropulsed laser modality that selectively destroys RPE cells followed by regeneration, and Thermal Stimulation of the Retina (TSR), a stimulative photothermal continuous wave laser modality that leads to an instant sublethal temperature increase in RPE cells, have shown therapeutic effects on Age-related Macular Degeneration (AMD) in mice. We investigate the differences between both laser modalities concerning RPE regeneration.

**Methods:**

For PCR array, 6 eyes of murine AMD models, apolipoprotein E and nuclear factor erythroid-derived 2- like 2 knock out mice respectively, were treated by neuroretina-sparing TSR or SRT. Untreated litter mates were controls. Eyes were enucleated either 1 or 7 days after laser treatment. For morphological analysis, porcine RPE/choroid organ cultures underwent the same laser treatment and were examined by calcein vitality staining 1 h and 1, 3 or 5 days after irradiation.

**Results:**

TSR did not induce the expression of cell-mediators connected to cell death. SRT induced necrosis associated cytokines as well as inflammation 1 but not 7 days after treatment. Morphologically, 1 h after TSR, there was no cell damage. One and 3 days after TSR, dense chromatin and cell destruction of single cells was seen. Five days after TSR, there were signs of migration and proliferation. In contrast, 1 h after SRT a defined necrotic area within the laser spot was seen. This lesion was closed over days by migration and proliferation of adjacent cells.

**Conclusions:**

SRT induces RPE cell death, followed by regeneration within a few days. It is accompanied by necrosis induced inflammation, RPE proliferation and migration. TSR does not induce immediate RPE cell death; however, migration and mitosis can be seen a few days after laser irradiation, not accompanied by necrosis-associated inflammation. Both might be a therapeutic option for the treatment of AMD.

## Background

Age related macular degeneration (AMD) is the most common cause for legal blindness in the industrialized world [1, 2]. The pathogenesis of AMD is multifactorial. Altered lipid metabolism [3–5], disturbed extracellular matrix homeostasis [6–8], inflammatory processes [9–11], and altered angiogenesis [12–14] are the four major pathways of AMD pathogenesis. Metabolites and cell-waste accumulate within and adjacent to Bruch’s membrane (BrM) [3], increasing BrM thickness and thereby diffusion barrier [15], inhibiting gas and nutrient exchange. Under the influence of external factors like smoking [16], Western diet [17] and oxidative stress [18, 19] as well as genetic predisposition [20], inflammatory processes appear, RPE cells and, consequently, photoreceptors degenerate leading to late stage dry AMD with patchy RPE/photoreceptor atrophy, called geographic atrophy. Pro-angiogenic factors may lead to choroidal neovascularization (CNV), forming fast progressive late stage neovascular (n)AMD. Both late-stage types of AMD are followed by vision deterioration.

Currently there is no treatment for early AMD, intermediate AMD, or geographic atrophy. Only nAMD can be treated by anti-vascular endothelial growth factor (VEGF) injections, mostly on a monthly schedule [21]. The need for treatment options for early and intermediate AMD is evident.

We could show that novel, sub-threshold laser therapies, thermal stimulation of the retina (TSR) and selective retina therapy (SRT), reduce pathologically thickened BrM and partially restore RPEs physiological morphology [22, 23]. Subthreshold is defined by its neuroretina sparing properties. No anatomical nor functional damage of neuroretina is produced. Both laser therapies might therefore be therapeutic options for early and intermediate AMD. TSR is a continuous wave laser irradiation therapy at 532 nm wavelength. To ensure stimulation and not coagulating, it is titrated to barely visible spots at the peripheral retina. Then power is reduced by 70% and laser spots are applied uniformly across the fundus. Thereby, spots are clinically invisible. TSR leads to a photothermal increase of temperature to an estimate of 45 °C [22]. Due to the lack of measurement possibilities in mice, this temperature was an estimate by Arrhenius integral calculation [22]. It induces no instant anatomical nor functional damage to neuroretina. It is still unclear, if it only leads to sublethal thermal increase in temperature within RPE or if it leads to a delayed cell damage resulting in delayed non-necrotic cell death. From our former studies, we postulate this hypothesis. We try to clarify this here. TSR has not been evaluated in human AMD and it has not been challenged in clinical trials. It is one of the subthreshold photothermal laser therapies, like subthreshold diode-micropulse laser (SDM) [24, 25], non-damaging retina therapy (NRT) [26], or subthreshold micropulse lasers (SML), like 577 nm wave length laser at 5 to 15% duty cycle [27–30]. These laser therapies induce a sublethal temperature increase in RPE cells, leaving neuroretina intact. SDM, SML and NRT have shown a beneficial effect on a variety of diseases, such as macular edema in diabetic retinopathy [30] and central serous chorioretinopathy [29]. Dosing of power remains an unsolved safety issue, especially since the intended region of treatment is the macula. It remains to be elucidated, whether the therapeutic benefits of these laser modi are based on fully sublethal temperature increase or on delayed RPE cell death followed by regeneration.

SRT is a micro pulsed laser irradiation therapy that has been applied at 527 nm, like R:GEN® by Lutronic [31–33], and at 532 nm wavelength, like the experimental laser system by Carl Zeiss Meditec [23, 34, 35]. In this study, we used the 532 nm experimental system, which has been adapted to our murine studies. For both systems, a train of 1.4 to 1.7 μs pulses at 100 Hz is produced to create a photodisruptive selective damage to RPE, leaving the neuroretina intact [23, 36]. This pulsed laser leads to the formation of micro bubbles around light absorbing melanosomes in RPE cells. Due to the pulsed design, temperature cannot evade melanosomes. Microbubbles lead to cell membrane rupture, thereby inducing cell death, without a temperature increase in surrounding tissues [36]. SRT is known for its beneficial effects in central serous chorioretinopathy and diabetic macular edema [31, 37]. Dosing of the energy level needed is a concern for macular treatment, but feedback-mechanisms have already been assessed in humans with promising results [32, 33]. It has not been evaluated in human AMD. A different photodisruptive laser therapy is nanopulsed retina rejuvenation therapy (2RT). This therapy has been evaluated in a clinical trial for the treatment of intermediate AMD. It failed its primary endpoint, but in a sub-analysis showed some beneficial effects in inhibiting AMD progression [38].

Recently, we evaluated the influence of both TSR and SRT on inflammatory mediators [39]. We could show that TSR initially acts anti-inflammatory and is followed by chemotactic processes. SRT, on the other hand, initially leads to an inflammatory response, most likely linked to the necrosis of RPE, followed by mild suppression of inflammatory mediators, like complement components, after a week. This led to the hypothesis that in SRT, RPE regeneration is the consequence of selective RPE necrosis. In TSR, RPE regeneration might be the consequence of delayed RPE cell death. This hypothesis is addressed here by the evaluation of cell-death linked cell-mediator expression in murine AMD models and by the evaluation of cell morphology in calcein stained porcine organ cultures.

## Materials and methods

### AMD mouse models

Both knock out AMD mouse models, Apolipoprotein (Apo)E knock out (−/−) and Nuclear factor erythroid 2-related factor 2 (NRF2) −/− have been described in detail [22, 40, 41]. ApoE −/−, NRF2 −/− and C57BL6/J control mice were purchased from the Jackson Laboratories (Bar Harbour, ME, USA). The homozygous genotype and screening for Crumbs homologue1 (CRB1) retinal degeneration (rd)8 mutation, known to interfere with the AMD phenotype of NRF2−/− mice [42], was confirmed by PCR from tail clips. Mice were kept on a regular 12 h night and day cycle and fed standard murine diet and water ad libitum. They were aged until 8 months of age for ApoE −/− and 9 months of age for NRF2 −/− mice. Both sexes were distributed equally. All animal experiments were conducted in accordance with the EU directive 2010/63/EU for animal experiments. They were approved by the animal ethics and welfare committee (approval number: V 242–7224.121-12 (61–5/14)) located at the ministry of energy transition, agriculture, environment and rural areas in Schleswig-Holstein according to German federal and European law. Animal experiments adhere to the NIH Guide for Care and Use of Laboratory Animals.

### Animal maintenance and anesthesia during experiments

All examinations and laser treatments were conducted under general anesthesia, like described before [22].

Anesthetized animals were placed on a rigid examination platform and body temperature was maintained within normal limits using a heating mat. Pupils were dilated and eyes were covered with a protective moisturizing gel. After examinations, the anesthesia was antagonized, like described before [22]. Anesthesia was uneventful in all mice. Animal wellbeing was evaluated by a standard score sheet and was uneventful in all included mice. After the final examination animals were euthanized by cervical dislocation at the day of enucleation under deep anesthesia.

### Examinations in mice

All examinations were conducted under general anesthesia. All mice were examined by funduscopy (MICRON III, phoenix research labs, Pleasanton, CA, USA), to assess integrity of retina, hallmarks of AMD (drusen-like retinal spots (DRS)), RPE atrophy and CNV.

Optical coherence tomography (OCT) (small animal OCT, thorlabs, Lübeck, Germany) was applied to evaluate retinal structure, confirm retinal integrity after laser treatment and to confirm CNV.

All examinations were repeated at the day of enucleation, thus 1 day or 1 week after laser treatment. Untreated controls also were examined twice, at inclusion and at enucleation day.

### Laser treatment in mice

For both SRT and TSR a frequency doubled Neodym-Vanadate (Nd:VO_4_) experimental laser (Carl Zeiss Meditec AG, Jena, Germany) with a wavelength of 532 nm was used. The light was coupled to an optical multimode fiber with a 70 × 70 μm^2^ core profile. The laser light was applied via contact laser-injector (Phoenix) attached to the Micron III camera. The pilot laser was controlled visually via live fundus imaging. Spot size was fixed to 50 μm in diameter.

For TSR duration of irradiation was fixed to 10 ms continuous wave mode. For SRT duration of irradiation was fixed to 300 ms, pulse-duration was ~ 1.4 μs at 100 Hz, creating 30 pulses per spot. Comparable to our previous work [22, 23], the intended effect was titrated visually by decreasing energy at the peripheral retina from a clearly visible white burn at higher energy to a barely visible spot at lower energy. The barely but instantly visible spot was classified as threshold of definite retinal burn/RPE destruction with visible neuroretinal involvement. Power was reduced by 70% to clearly avoid neuroretinal damage and to ensure neuroretina-sparing temperature increase for TSR (power range: 4 mW ± 1.7), or an RPE-selective laser damage for SRT (energy range: 2.5 μJ ± 0.9) respectively. The invisible 50 μm TSR/SRT laser spots were distributed uniformly across the retina at 1 spot interspot spacing to an optic disc centered approx. 50° field of view. No laser spot was applied to vasculature or the optic disc. This method is an established and validated method for murine TSR or SRT treatment [22, 23].

### PCR array

RT^2^ profiler PCR array (Qiagen®, Frederick, Maryland, USA; Mouse Inflammatory Response & Autoimmunity; PAMM-077Z) was used to determine regulation of inflammation and cell-death related mediators of TSR or SRT treated eyes in comparison with untreated littermates in AMD mouse models. From 84 analytes that are included in our array, those 12 genes relevant for the examination of cell death and regeneration were included in the study. The procedure was described in detail before [39]. Briefly, for RNA isolation, posterior cups were homogenized and total RNA was isolated using TRI Reagent® according to the manufacturer’s instructions.

Isolated RNA was converted to cDNA using the RT^2^ First Strand Kit (Qiagen). The mixture was aliquoted (25 μl) to each well of the same RT^2^ Profiler PCR Array plate (96-well plate) containing the pre-dispensed gene-specific primer sets. PCR was performed using a 7500 Fast Real Time cycler (Applied Biosystems).

Qiagens online Web analysis tool (Gene globe) was used to calculate the fold change by determining the ratio of mRNA levels to control values using the Δ threshold cycle (Ct) method (2^−ΔΔCt^). All data were normalized to the housekeeping genes of PAMM-077Z panel (Quiagen), which were: beta Actin, beta-2 Microglobulin, Glyceraldehyde-3-phosphate Dehydrogenase, beta Glucuronidase and Heat Shock Protein 90 alpha (cytosolic), class B member 1. PCR conditions used: hold for 10 min at 95 °C, followed by 40 cycles of 15 s at 95 °C and 60 s at 60 °C.

### Porcine organ cultures preparation

Fresh porcine eyes were acquired from a local abattoir. The preparation has been described in detail elsewhere [43]. Briefly, the eye bulbs were cut at the limbus removing the anterior segment, including lens and vitreous body. Eyes were opened by longitudinal incisions and neuroretina was removed. The complex of RPE, BrM and choroid was removed carefully from sclera. A plastic ring-system was inserted, and the RPE/BrM/choroid complex fixed to it. Rings were placed into 12-well-culture plate and kept warm at 37 °C in 1.5 ml organ culture medium (see Richert et al. [43]).

### TSR and SRT in organ cultures

Organ cultures were placed under a slit-lamp adapted laser system in organ culture medium in 12-well plates. Organ cultures were irradiated by either TSR (100 ms duration, 200 μm spot size, power titrated to no instant cell-death and a cell death rate of ~ 2% 1 day after TSR), or by SRT (300 ms duration, 100 Hz, 1.4 μs pulse duration, 200 μm spot size, energy titrated to an initial cell-death rate of ~ 80% in). Calcein-assays were performed afterwards to confirm cell-death rates. This method was also applied to check the quality of organ cultures [44]. For titration, preliminary examinations were needed. Four organ-culture rings each laser mode were used. Microscopically visible marker spots, to enable orientation within a field of partly invisible laser spots, were placed in 4 quadrants of the organ cultures. TSR/SRT laser spots at different power/energy levels were applied within the marker lesions. Then cell death rate was calculated semi-automatically by Axio-Vision (Zeiss, Jena, Germany) after calcein staining. A cell death rate of ~ 2% was achieved at 25 mW 1 day after TSR. For SRT, an initial cell death rate of ~ 80% was achieved at 180 mJ/cm^2^. The titrated power/energy level was then used for the main experiment.

### Calcein assay

Calcein assays were conducted to examine integrity and vitality of RPE organ cultures. Calcein fluoresces if cleaved by active enzymes integrated into vital RPE cell membranes. Dead cells do not fluoresce, since enzymatic cleavage does not function in dead cells [45]. Organ culture explants were incubated in 2 ml culture medium with 4 μg/ml Calcein at 37 °C for 45 min. Afterwards they were rinsed twice in phosphate buffered saline. Cell vitality was measured by fluorescence microscopy (Axiovert 100, Zeiss, Jena, Germany) at λex/λem = 497/517 nm and documented photographically.

### Statistics

#### Gene expression by PCR arrays

Fold changes in gene expression for pairwise comparison using the ΔΔCT method was calculated through Qiagen® Web analysis tool and *p*-values were provided, at a confidence interval of 95% and a type-1 error of 5%.

For comparison of TSR or SRT treated to untreated eyes, one randomized eye of ApoE−/− or NRF2−/− was treated by TSR or SRT. One day or 1 week after treatment these eyes were compared to entirely untreated age-matched randomized control eyes of the same genotypes in groups of 6 eyes each.

#### Determination of cell size and number by calcein assays

For determination of cell death and regeneration, 1 h, 1, 3 and 5 days after TSR or SRT laser treatment, calcein assay photographs were analyzed semiautomatically by AxioVision (Zeiss, Jena, Germany). Cell size and number of vital cells within the defined 200 μm laser spot were measured and noted for statistical analysis. The median percentage of non-fluorescent area within the defined laser spot was calculated from 12 spots each. These spots derived from 4 organ culture rings (OCR) each laser modus.

## Results

### In-vivo imaging

All mice showed certain signs of AMD, such as drusen-like retinal spots (DRS), RPE pigmentation irregularities and mottling. CNV or geographic atrophy, as markers for late AMD, were not seen in any mouse. AMD disease grading, like explained before [42] (1 = physiological retina, 2 = 1–14 DRS, 3 = 15–100 DRS, 4= > 100 DRS, 5 = any number of DRS plus signs of late AMD), revealed a mean of grade 2.6 +/− 0.6 in NRF2−/− and 1.7 +/− 0.6 in ApoE−/− mice. Laser treatment did not significantly alter the AMD grade of either NRF2 −/− or ApoE −/− mice. There were no signs of neuroretinal damage in fundus examination or in OCT after either laser treatment.

### PCR-Array for the expression level of inflammatory cell mediators linked to apoptosis and necrosis

Table 1 shows the results of PCR array-based analysis of cell mediator expression.

**Table 1:** Single values of examined apoptosis and necrosis linked inflammatory cell mediators

Gene	ApoE								NRF2							
	TSR 1d		TSR 7d		SRT 1d		SRT 7d		TSR 1d		TSR 7d		SRT 1d		SRT 7d	
	fold	*p*	fold	*p*	fold	*p*	fold	*p*	fold	*p*	fold	*p*	fold	*p*	fold	*p*
FasL	1.2		1.8		1.7		−2.5		−1.5		1.1		−1.3		1.3	
IFNg	**−5.1**	< 0.01	1.6		−1.9		− 1.3		1.1		1.5		**2.3**	< 0.01	1.9	
Il1b	−1.4		1.1		3.3		−1.7		−1.8		−1.8		−5.2		**−2.0**	0.02
Il18	1.4		1.1		1.6		−1.5		−1.6		1.1		−1.1		−1.3	
NFkb1	−1.3		−1.6		**5.0**	< 0.01	1.3		−1.2		1.1		1.2		−1.7	
C3	−1.9		1.1		**4.0**	0.01	− 1.4		−1.2		−1.8		1.2		−3.1	
Tlr1	−1.2		1.6		2.1		− 1.7		−1.7		1.1		**2.5**	0.04	−1.4	
Tlr2	−1.8		−1.1		**4.1**	< 0.01	−1.3		−1.9		1.2		−1.1		−1.1	
Tlr4	−1.5		1.1		**2.8**	0.01	−1.1		**−2.0**	< 0.01	−1.1		1.5		−1.5	
Tlr7	−1.3		1.6		1.9		−2.3		**−2.3**	0.05	1.7		1.2		−1.4	
Tlr9	−1.7		1.1		**4.0**	< 0.01	−1.5		**−3.0**	< 0.01	−1.6		−1.6		−1.4	
Tnfsf14	**−3.5**	< 0.01	2.6		1.1		−3.0		1.9		2.5		−2.5		1.3	

Column 1 shows the name of the gene examined. For each genotype, x-fold expression in the treated eyes (TSR or SRT respectively) compared with untreated eyes and their p-values are given. FasL, Ifng, Il1b, Il18, Nfkb1 may be linked to apoptosis (bold frame). C3, Tlr1, 2, 4, 7, 9 and Tnfsf14 may be linked to necrosis

Cell-death-linked apoptotic factors, like Fas ligand (FasL), Interferon gamma (IFNg), Interleukin (IL)1 beta and IL18, as well as Nuclear factor kappa light-chain enhancer of activated B-cells (NFκb) were examined. FasL was not altered neither by TSR nor by SRT, 1 or 7 days after laser treatment. IFNg was downregulated by TSR, 1 day after treatment in ApoE−/− and upregulated 1 day after SRT in NRF2−/− mice. IL1b was downregulated 7 days after SRT in NRF2−/− mice. IL18 was not altered in either treatment. Expression of NFκb1 was increased 1 day after SRT.

Necrosis-linked factors were also examined. Complement system centered complement factor 3 (C3) expression, was increased 4-fold, 1 day after SRT in ApoE−/−. Toll-like receptors (Tlr) were increased 1 day after SRT in both models. In NRF2 −/− mice Tlr expression was decreased 1 day after TSR. Tumor necrosis factor superfamily (Tnfsf) was downregulated 1 day after TSR in ApoE −/− mice.

### Calcein assay

**Fig. 1:**
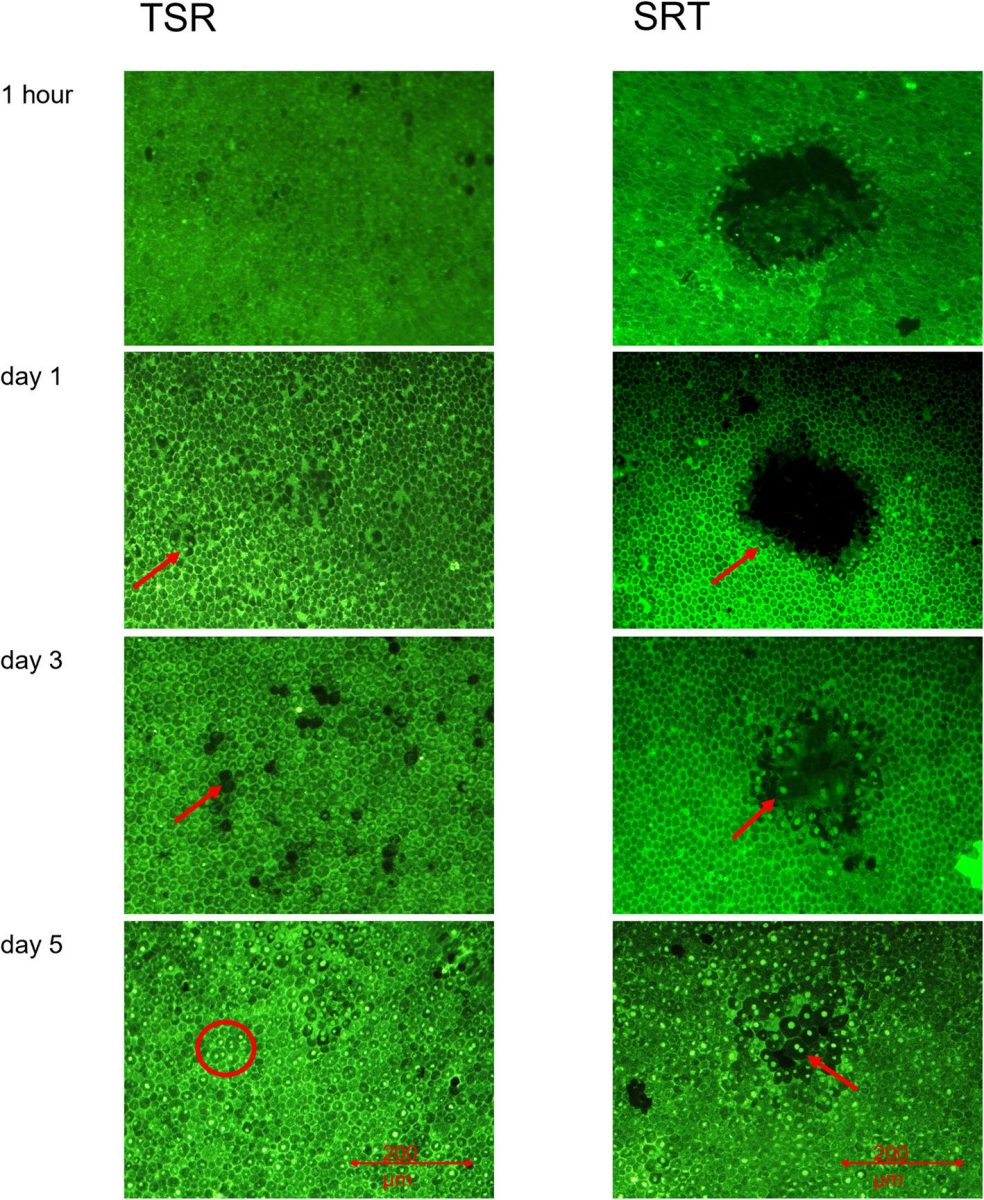
Representative examples of calcein assays to determine cell vitality and proliferation at the different given time points after TSR and SRT. TSR is followed by scattered changes like the appearance of condensed cell nuclei (red arrow). Empty spaces indicating cell death are mostly seen at day 3 (red arrow) and rarely detected at day 5. Mitosis figures (red circle), indicating regeneration can be seen at day 5. SRT induced instant RPE cell-death, followed by regenerative processes like condensed cell nuclei (red arrow, day 1) migration (red arrow, day 3) and proliferation (red arrow, day 5) at the rim of laser lesions until lesion closure

In porcine organ cultures RPE cell vitality was examined by calcein assay. In addition, regeneration processes could be examined. Figure 1 displays calcein assays after TSR and after SRT at 200 μm spot-size 1 h, 1, 3 or 5 days after laser irradiation. There was no instantly visible cell damage 1 h after TSR. TSR-treated organ cultures showed condensed nuclei from day 1 (2% cell death ±4.3% within the lasered area, *n* = 12 spots, 4 organ culture rings [OCR]). From day 3 (mean cell damage 4.2% ± 3.6 within the lasered area, *n* = 12 spots, 4 OCR), regenerative signs, like cell migration and cell proliferation were seen. Cell replacement and lesion closure were seen in small patches across the spot. Lesion closure was achieved at day 5. SRT induced instant cell necrosis covering the whole spot, followed by proliferation and migration from day 1 after laser irradiation (mean area of cell death 60% ± 36.5 *n* = 12 spots, 4 OCR). At day three 6.7% (± 6.5%, *n* = 12 spots, 4 OCR) of the initial spot area were not filled with new cells. Lesion closure was complete after 5 days.

## Discussion

AMD is a multifactorial disease composed of an altered lipid metabolism, changed extracellular matrix, inflammatory processes and mislead angiogenesis. There is no pathogenesis-driven therapy that targets all the above-named aspects of AMD. Current therapeutic strategies aim at certain parts of AMD pathogenesis. To date, only the treatment of pathologic angiogenesis by anti-vascular endothelial growth factor (VEGF) antagonists [21] in neovascular AMD has shown great therapeutic benefit. A therapy for early or intermediate AMD has yet to be developed. We know that TSR, as well as SRT have therapeutic effects on AMD-like alterations in AMD mouse models. Thickened BrM becomes thinner and pathologically altered RPE becomes a more physiological phenotype [22, 23]. BrM restructuring, also shown by others after nanopulse laser treatment in an AMD mouse model [46], aims at extracellular matrix and is mediated by an increase in matrix metallo-protease (MMP) expression, especially active MMP-2 [43, 47]. However, RPE regeneration may have a positive influence not only on extracellular matrix.

As for inflammation, it is more likely that inflammatory processes are altered in short term due to the laser impact on the treated RPE cells. RPE cells are reduced in viability if put under constant pro-inflammatory stress [48]. This condition can be found in AMD mouse models, like ApoE −/− mice [39]. We could show earlier that TSR suppresses inflammatory processes 1 day after treatment and is followed by chemotaxis 1 week after laser irradiation. SRT induces inflammation instantly due to the intended necrosis. Inflammatory processes are unaltered or even suppressed 1 week after laser irradiation [39]. A lasting therapeutic effect that derives from suppression of pro-inflammatory processes over a long time, useful for the treatment of AMD, has not been shown so far.

The effect on lipid metabolism has not been evaluated yet. It should be part of future studies to increase the understanding of the way of action of both TSR and SRT.

The effect of TSR and SRT on neovascular AMD has also not been evaluated in a translational model. We know from organ culture experiments that both TSR and SRT lead to a reduction of VEGF expression and increase of PEDF expression [43, 49]. However, both laser modi have shown to influence more than only one aspect of AMD pathogenesis in AMD mouse models. Both may be therapeutic options for the treatment of AMD, yet to be evaluated in clinical trials.

LEAD study was a prospective, sham-controlled, double-blinded study of photodisruptive nanopulsed laser therapy “2RT” for the treatment of intermediate AMD. Nanopulsed 2RT can be regarded as similar to micropulsed SRT, although technical and safety profiles differ. LEAD study revealed retinal bleeding in ~ 7% of treated eyes, which is a concern regarding therapy within macula. This has not been reported for SRT. LEAD was unsuccessful in terms of achieving the primary endpoint, the inhibition of the conversion of intermediate to neovascular AMD [38]. A retrospective analysis, however, dividing intermediate AMD into reticular pseudodrusen (RPD) AMD and non-RPD AMD showed that 2RT leads to delayed conversion of intermediate AMD to neovascular AMD in patients with non-RPD AMD. In patients with RPD AMD laser treatment even accelerated disease conversion. The study demonstrated that patient selection and a good understanding of the molecular mechanisms of a new treatment are crucial. LEAD study was, the only “subthreshold-laser for the treatment of dry AMD” study so far that was conducted in a prospective, sham-controlled, double-blinded manner. For any clinical evaluation of SRT or TSR for the treatment of dry AMD, a prospective, sham-controlled, blinded approach is crucial.

Photothermal stimulation by subthreshold diode laser micropulse (SDM) therapy, somewhat comparable to TSR, has been evaluated for the treatment of geographic atrophy recently [25]. SDM differs in laser mechanism. It is achieved by the so-called duty cycle. Within a single spot, laser is turned on 5% of the time and turned off 95% of the time in a pulsed manner. Thereby heat can hardly evade RPE, creating a somewhat RPE selective photothermal effect [50]. The geographic atrophy study design was retrospective and uncontrolled. Data should be evaluated cautiously, but apparently SDM may slow the progression of RPE atrophy [25]. Subthreshold photothermal laser modi are in discussion for the treatment of intermediate or early AMD, but a clinical trial, like LEAD study, has not been conducted yet and therefore it remains unclear if these laser therapies are an option for the treatment of early and intermediate AMD.

A better understanding of the molecular way of action of subthreshold laser therapies is needed to enable a good patient selection and reach the final goal of maybe finding a novel treatment for AMD.

### So how do TSR and SRT act?

**Fig. 2:**
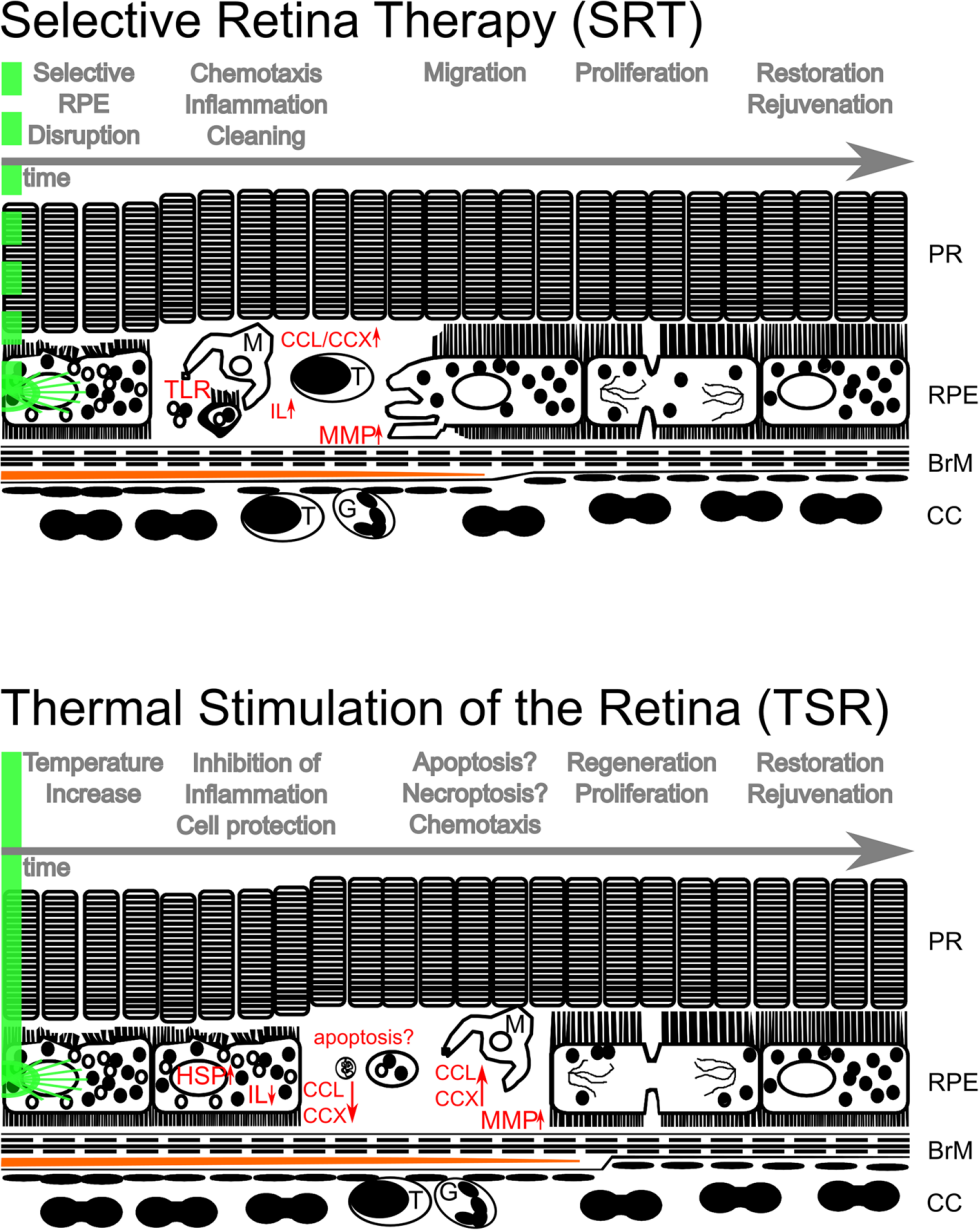
SRT and TSR presumed way of action. SRT (above) induces initial necrosis by photodisruption of RPE. Toll-like receptors (TLR) are expressed. Microglia (M) are attracted, interleukins (IL) and chemokines (CCL/CCX) are increased in expression thereby attracting more cells of the immune system. Destroyed cells are removed and neighboring RPE starts migration and proliferation to close the lesion. Increased matrix metallo-proteases (MMP) restructure Bruch’s membrane (BrM) and intra-BrM lipids (orange line) are removed. A rejuvenated, restored BrM/RPE complex is achieved thereafter. Neuroretina stays intact during this process. TSR (below) does not induce necrosis. It initially increases temperature within RPE leading to cell protective mechanisms, like the expression of heat shock proteins (HSP). Inflammatory processes, like interleukin expression and chemotaxis are initially suppressed. We hypothesize that single RPE cells die later and are instantly removed. This presumably apoptotic process, since cell death and replacement are seen in absence of inflammation, is accompanied by migration and proliferation of RPE cells. MMP expression is increased leading to BrM remodeling and removal of accumulated lipids (orange line). A rejuvenated restored BrM/RPE complex is achieved without damage to neuroretina. PR (photoreceptors), G (granulocytes), T (T-lymphocytes)

Based on findings from this paper, as well as findings from former publications [22, 23, 39, 43, 49, 51], we propose the following hypothetical model, as depicted in Fig. 2.

SRT, as expected, leads to defined necrosis of RPE cells [36] (see Figs. 1 and 2 and Table 1). The photo-disruptive effect induces RPE cell death that does not harm neuroretina [23, 52]. Necrosis is accompanied by inflammation and chemotaxis to remove cell-debris [39]. RPE regenerates [43] and active MMP expression is increased, leading to thinning of the pathologically thickened BrM and a conversion of pathological to physiological RPE in AMD [23, 53]. Restoring and rejuvenating RPE could be a useful therapeutic approach to treat intermediate AMD. VEGF expression is reduced and PEDF expression increased, thereby preventing neovascular processes [43].

Thermal increase (like in TSR (see Fig. 2)), on the other hand, leads to various cell protective mechanisms like HSP expression [54, 55]. It may additionally lead to a delayed small scale RPE cell-death (see Fig. 1), as shown by others [55] and proposed and shown by us here. The spotty pattern of cell death within the irradiated area (see Fig. 1) most likely depends on the temperature applied. Up until now, there is no device to keep the temperature elevation precisely at the needed level. The therapeutic window is possibly very small. Overtreatment would lead to instant full scale or delayed large scale RPE cell death at the laser spot and likely to neuroretinal damage. If applied at the intended temperature level, this level is not known yet, RPE cells are stimulated, no instant cell death occurs and neuroretina is undamaged. We hypothesize that a few cells die in a non-necrotic way, then followed by immediate replacement [49], without inflammation [39] and, like SRT, without neuroretinal damage [22]. The regenerative process [22] is accompanied by an increase in active MMP expression [49], also leading to thinning of BrM. VEGF antagonistic PEDF is overexpressed, possibly also preventing neovascular processes [49]. It remains a hypothesis that TSR leads to delayed non-necrotic cell death, but evidence is there and shown here. It is a less invasive, less immunogenic approach. If that is an advantage or disadvantage for AMD treatment is unclear. It possibly has fewer side effects and might therefore be a preventive option for AMD.

From the presented data and from what we have known so far, one cannot decide if either TSR or SRT are the better therapeutic option for the treatment of a certain type of AMD. More needs to be known about the way of action to better attribute either laser modus to a certain type of AMD.

The above shown data is limited by small numbers of eyes and methods to differentiate between the different regenerative properties. However, the differences between both laser modi become clearly evident. SRT leads to necrosis followed by regeneration. TSR does not induce instant cell death. The sublethal temperature increase is initially not lethal but stimulating. It might lead to delayed RPE cell death in absence of inflammation, followed by regeneration. A presumed apoptotic process needs to be looked at closer in future studies. Miura et al. could show, in an in-vitro approach of cultured RPE cells, that delayed, apoptotic cell death occurs at temperatures of 46 to 48 °C. It was not seen below 44 °C. Necrosis was seen above 50 °C [55]. Although the model was different and irradiation time much longer (10 s), it is the only manuscript, as far as the authors are aware of, that gives an idea of the needed temperature to achieve the desired effect. From our previous studies we estimate an achieved temperature of about 45 °C in TSR treated murine RPE [22]. Thus, we treat somewhere between stimulation and apoptosis induction. 45 °C are an estimate and have not been validated by an invasive or non-invasive temperature measurement so far, because there is no in-vivo real-time temperature measurement method for mice known to the authors. The needed temperature to achieve the desired therapeutic goal is unclear. At what temperature do we see an undertreatment? All this needs to be part of future studies.

Like all subthreshold laser modi, TSR is a very temperature dependent laser mode, and the therapeutic window is small. Without an exact real-time temperature measurement device, an exact validated temperature cannot be achieved in this model. Also, intraindividual differences in pigmentation across the fundus and variable optic quality in different fundus areas do not allow for an exact dosing of the applied laser power. One would need an optoacoustic or reflectometric feedback device to provide for a real-time temperature estimate that comes close to the real achieved temperature of every single laser spot within RPE [56, 57]. Such a device does not exist for mice. Our method of TSR in mice is, however, as good as it gets. We are currently working on a project to design an optoacoustic feedback system for mice to enable dose-response examinations in the future. At present, this is unfortunately not possible.

Concerning the expression of the presented 12 genes relevant for cell death and regeneration after laser treatment, evidence is good. The presented data derives from an individual experimental group of mice, which were part of an experimental series of a different project. These mice reproduce the data shown before [39]. However, our given numbers would most certainly vary, if the experiment was to be repeated now, with a different breed at a different time, treated by different researchers. The exact fold-change numbers themselves are not so relevant, it is more relevant to perceive the statistical significance of differences in gene expression. PCR arrays give an estimate of the genes that might be involved in certain processes. Further research on protein expression is needed, then with a focus on those proteins coded by the overexpressed RNA we detected here. A novel and entirely independent experiment that includes more data on typical genes for cell death and regeneration would enhance the accuracy of our findings and enable a better differentiation between the two laser modi.

We intentionally did not compare the treated eye with fellow untreated eyes. It has been shown by other groups and our group that laser treatment of one eye, also leads to changes in gene expression and BrM thickness within the fellow eyes [22, 23, 46].

Generally, the expression of genes needs to be judged carefully, since gene expression does not necessarily mean protein secretion.

Despite uncertainties concerning laser therapy for early and intermediate AMD it is reasonable to consider both SRT and TSR potential therapeutic means for AMD. Both need to be evaluated in humans. Study populations need to be selected carefully, considering the different grades and forms of AMD. Hypothetically, TSR, due to its anti-inflammatory and small-scale regenerative properties, might be a preventive or therapeutic option for early AMD with small drusen. SRT, due to its large scale RPE regenerative/rejuvenating properties might be a better option for intermediate AMD. Future studies, especially human studies, will have to determine the benefit of TSR and SRT for the treatment of early and intermediate AMD.

## Data Availability

The datasets used and/or analysed during the current study are available from the corresponding author on reasonable request.

## Abbreviations

AMD: Age-related macular degeneration
ApoE −/−: Apolipoprotein E knock out
BrM: Bruch’s Membrane
C3: Complement factor 3
CNV: Choroidal neovascularization
CRB1: Crumbs homologue gene 1
DRS: Drusen like retinal spots
FasL: Fas ligand
IFNg: Interferon gamma
IL: Interleukin
MMP: Matrix metallo proteinase
nAMD: Neovascular age-related macular degeneration
NFκb: Nuclear factor kappa light-chain enhancer of activated B-cells
NRF2 −/−: Nuclear factor erythroid 2-related factor 2
NRT: Non-damaging retinal laser therapy
OCT: Optical coherence tomography
RPE: Retinal pigment epithelium
SDM: Subthreshold diode-laser micropulse
SML: Subthreshold micropulse laser
SRT: Selective Retina Therapy
Tlr: Toll-like receptor
Tnfsf: Tumor necrosis factor superfamily
TSR: Thermal Stimulation of the Retina
VEGF: Vascular endothelial growth factor
2RT: Retinal Rejuvenation Therapy
